# Crowdfunding scientific research: Descriptive insights and correlates of funding success

**DOI:** 10.1371/journal.pone.0208384

**Published:** 2019-01-04

**Authors:** Henry Sauermann, Chiara Franzoni, Kourosh Shafi

**Affiliations:** 1 ESMT Berlin, Berlin, Germany; 2 National Bureau of Economic Research, Cambridge, Massachusetts, United States of America; 3 School of Management, Politecnico di Milano, Milan, Italy; 4 University of Florida, Gainesville, Florida, United States of America; Utrecht University, NETHERLANDS

## Abstract

Crowdfunding has gained traction as a mechanism to raise resources for entrepreneurial and artistic projects, yet there is little systematic evidence on the potential of crowdfunding for scientific research. We first briefly review prior research on crowdfunding and give an overview of dedicated platforms for crowdfunding research. We then analyze data from over 700 campaigns on the largest dedicated platform, Experiment.com. Our descriptive analysis provides insights regarding the creators seeking funding, the projects they are seeking funding for, and the campaigns themselves. We then examine how these characteristics relate to fundraising success. The findings highlight important differences between crowdfunding and traditional funding mechanisms for research, including high use by students and other junior investigators but also relatively small project size. Students and junior investigators are more likely to succeed than senior scientists, and women have higher success rates than men. Conventional signals of quality–including scientists’ prior publications–have little relationship with funding success, suggesting that the crowd may apply different decision criteria than traditional funding agencies. Our results highlight significant opportunities for crowdfunding in the context of science while also pointing towards unique challenges. We relate our findings to research on the economics of science and on crowdfunding, and we discuss connections with other emerging mechanisms to involve the public in scientific research.

## Introduction

Crowdfunding–an open call for money from the general public–has become an important source of funding for entrepreneurial, artistic, and social projects [1–4]. More recently, scientists and policy makers have suggested that crowdfunding could also be valuable to support scientific research [5–7] and some universities actively encourage their researchers to start crowdfunding campaigns [8]. The public discussion as well as related work on crowdsourcing and Citizen Science suggest several potential benefits [9–11]. One hope is that funding from the crowd can expand the total amount of resources available for science, or at least partly compensate for tighter budgets of traditional funding agencies [6]. In light of the increasing difficulties especially junior scientists face in getting funding through traditional channels [12], some observers highlight that the crowd may be more willing to fund researchers who do not yet have an established track record [7]. Finally, “broadcasting” proposals to a large number of potential funders may allow researchers to identify those supporters who share an interest in the same topics, even if these topics are not mainstream or priorities for traditional funding agencies [10, 13].

Despite these hopes, however, the potential of crowdfunding for scientific research is not clear. Many crowdfunding campaigns in other domains fail, suggesting that raising money from the crowd can be quite challenging [2, 14]. Moreover, research projects have characteristics that would be expected to make it challenging to raise funding from the crowd. Among others, scientific research is often risky, while members of the crowd may have a preference for projects that are likely to succeed [15]. Similarly, there is an asymmetry in the knowledge of highly trained scientists and potential “citizen” funders, such that the latter may find it difficult to assess the quality and merit of research proposals [15, 16]. Research projects also cannot typically offer the tangible outputs that are often “pre-sold” on general-purpose platforms such as Kickstarter, and scientific research projects may generally be perceived to have less direct use value than other types of projects [15, 17]. On the other hand, crowdfunding platforms that specialize in scientific research projects may attract backers with different kinds of motivations and decision criteria than general-purpose platforms. Moreover, they may be able to offer tools that are tailored to the needs of scientists and their funders and may help increase the odds of fundraising success.

To assess the potential of crowdfunding for scientific research, we report initial evidence from Experiment.com, the currently largest dedicated platform for crowdfunding research. We first provide descriptive information on the creators seeking funding, the projects they are seeking funding for, and features of the crowdfunding campaigns. We then investigate how these various characteristics are related to campaign success. We compare the results to prior research on the predictors of fundraising success in crowdfunding but also to research on traditional scientific funding mechanisms such as government grants. Finally, we examine whether and how predictors of crowdfunding success differ from those that predict attention from a more professional audience–journalists covering scientific research.

Our analysis provides new evidence on the state of crowdfunding in scientific research and should be of interest to social scientists as well as to scientists who consider starting their own crowdfunding campaigns. By providing empirical evidence from the specific context of science, this study also contributes to the broader literature on crowdfunding, which tends to focus on general-purpose platforms.

## Prior research

Although prominent success stories such as the Pebble Watch or the Oculus Virtual Reality Headset have demonstrated the potential of crowdfunding, many campaigns fail to reach their funding targets [2, 14]. As such, a growing literature in fields as diverse as economics, management, and the natural sciences has started to examine crowdfunding from a descriptive perspective, and to explore potential drivers of fundraising success [18]. Most of these contributions, however, have looked at crowdfunding for startups, technology development, or projects in the arts or cultural industries. In contrast, there is little evidence on the potential of crowdfunding as a tool to raise resources for scientific research [17, 19].

Even though a unified framework for studying crowdfunding has not emerged yet [20], most of the prior literature examines how crowdfunding success relates to factors in the following three broad domains.

First, studies have examined how fundraising success is related to certain characteristics of the individuals who are seeking to raise funding (i.e., the “creators” of a campaign). In particular, several studies have explored gender differences in funding success, finding that female creators, or teams that have at least one female creator, are more likely to achieve success compared to male creators [21–23]. Other studies have considered creators’ broader social networks, highlighting the role of the social interconnectedness of the creator in explaining funding outcomes [2, 23–25]. Related work has considered the geographic location of creators, suggesting that crowdfunding can provide better access to funding for creators in less central locations and lead to more distributed funding outcomes than traditional mechanisms [26, 27].

Second, fundraising success is related to characteristics of the project, i.e., what funding is raised for. The existing evidence suggests that projects with non-profit goals are more likely to be funded than projects with for-profit goals [28, 29]. Moreover, there is robust evidence that projects with smaller budgets are more likely to achieve their targets [2, 23, 24]. Recent work on both rewards-based and equity crowdfunding suggests that more radical and innovative projects are less likely to be funded, perhaps reflecting that backers doubt the feasibility of radical proposals or that radical proposals appear less useful in addressing currently perceived needs [15, 30].

Third, attention has been directed at the link between crowdfunding success and features of the campaign itself, e.g., what information is presented, how it is presented, and how creators interact with the crowd. Research has found that the amount of information provided about a project is positively correlated to funding success [23, 25], particularly when the information makes the project more understandable and relatable to the crowd [31]. Information given in a visual form, including videos, is particularly useful [2, 23, 32]. Project updates during the campaign can further increase the likelihood of success [33]. Endorsements by a third party, such as business angels or venture capitalists, correlate positively with fundraising success, perhaps because they serve as a signal of quality and reduce the information asymmetry between the creator and the crowd [34, 35]. Finally, a study in the context of scientific research suggests that campaigns were more successful when scientists started nurturing an audience for their projects before the crowdfunding campaign, taking advantage of their social networks [19].

We build on this existing work to provide insights into crowdfunding campaigns in an understudied context–scientific research. In considering specific factors within each of the three domains, we can thus also draw on prior research in the economics of science, including work on predictors of fundraising success in the traditional (grant-based) system. With respect to creator characteristics, for example, we distinguish junior versus senior researcher status as well as academic versus industry affiliations [36, 37]. Similarly, we classify projects based on their research objectives, develop a proxy for the degree to which creators describe a project as risky, and examine what kinds of research expenses creators plan to cover with the funding raised [38–40]. For campaign characteristics, we consider a range of factors such as “lab notes”, as well as the listing of prior publications, which are often taken as signals of quality by traditional scientific funding agencies [36].

## Crowdfunding platforms for scientific research projects

Our data come from the platform Experiment.com, which is dedicated to crowdfunding for scientific research. This US-based platform was established in May 2012 under the name Microryza and was later renamed. The platform allows investigators to create a profile and run a campaign to raise funding for a research project. Experiment.com pre-screens campaigns to ensure minimum standards regarding clarity of the research question, transparency, and researcher expertise [41]. Upon launch, a campaign stays open for a limited amount of time, typically 30–45 days. Campaigns are governed by an “all-or-nothing” rule, i.e., donors are charged and pledged funds transferred to the campaign creators only if the stated funding goal is reached. In this sense, campaigns resemble the all-or-nothing nature of competitive grant proposals made to traditional funding agencies.

There are several other platforms for crowdfunding scientific research, following a similar model as Experiment.com. Table 1 provides examples of other relevant platforms, with basic information such as the founding date and the number of projects hosted. The table shows that some of these platforms are independent, while others are run by universities or funding agencies primarily for their own purposes. While some have been operating for several years, others have failed. Experiment.com is, to the best of our knowledge, the largest dedicated platform for the crowdfunding of scientific research projects.

**Table 1:** Examples of dedicated platforms for crowdfunding scientific research.

Name	URL	Opened	Status as of January 2018
***Independent platforms***			
Experiment	https://www.Experiment.com	2012	Active. 1,820 projects hosted.
Petridish	http://blog.petridish.org/	2012	Closed. 32 projects hosted.
Davincicrowd	http://www.davincicrowd.com	2012	Active. 92 projects hosted.
Consano	http://www.consano.org	2013	Active. 67 projects hosted.
Donorscure	http://www.donorscure.org	2013	Active. 16 projects hosted.
Wallacea/Crowdscience	http://crowd.science	2014	Active. 36 projects hosted.
Futsci	http://futsci.com	2015	Active. 12 projects hosted.
Science Starter	http://www.sciencestarter.de	2015	Active. 122 projects hosted.
***Institution-specific platforms***			
Cancer Research UK	http://myprojects.cancerresearchuk.org	2008	Closed.
Georgia Institute of Technology	https://www.gatech.edu/	2013	Closed.
UCLA	http://spark.ucla.edu	2014	Active. 15 projects hosted.
Virginia Tech	http://crowdfund.vt.edu	2017	Active. 29 projects hosted.

Crowdfunding platforms such as Experiment.com should be distinguished from two other types of platforms available to researchers. First, there are charity fundraising platforms such as Benefunder and Thecommongood. Such platforms differ from Experiment.com in that funds are typically raised for an organization or general cause rather than specific research projects, fundraising is open ended with no time limit, and there is no explicit fundraising target and no “all-or-nothing” mechanism. Thus, these platforms are similar to traditional charity institutions, except that they use the online channel for fundraising. Second, there are general-purpose “reward-based” platforms, such as Kickstarter or Indiegogo. These platforms are project-based and follow an all-or-nothing model, but they are primarily for business or artistic projects and rarely host campaigns that focus on scientific research. They usually require creators to give rewards to the backers and have other specific provisions that make the fundraising for scientific research projects difficult. For example, Kickstarter explicitly excludes projects aimed at the treatment or prevention of illnesses [42] and Indiegogo temporarily stopped accepting non-profit projects in February 2018 [43].

## Data and measures

We obtained from Experiment.com leadership the links to all crowdfunding campaigns that were started since the platform launch in May 2012 and for which success or failure status was known in August 2015. We scraped the webpage content of these campaigns to obtain measures for a wide range of project characteristics as well as funding outcomes. We hand-coded additional variables based on project descriptions on the campaign webpages and profile pages of campaign creators. We received written permission from Experiment.com to collect these data.

We dropped from the analysis 16 campaigns whose webpages are incomplete. Our final sample includes 725 campaigns. Of these campaigns, 68% were started by a single creator. The remaining campaigns were posted by teams ranging from 2 to 7 creators, for a total of 1,148 creators in our sample.

In the following, we describe variables and measures. Tables 2 and 3 show summary statistics at the level of individual creators and of campaigns, respectively. S1 Table shows selected correlations.

**Table 2:** Summary statistics at the creator level.

		1 All creators N = 1,148	2 First listed N = 725	3 Team: first N = 230	4 Team: not first N = 423	3–4
**Affiliation**	Educational institution	0.81	0.80	0.83	0.81	ns
	Firm	0.05	0.05	0.04	0.03	ns
	Other organization	0.08	0.08	0.10	0.08	ns
	None/independent	0.05	0.06	0.03	0.04	ns
**Position**	Below PhD/MD	0.24	0.21	0.17	0.29	**
	PhD/MD	0.20	0.23	0.21	0.15	+
	Postdoc	0.06	0.05	0.08	0.07	ns
	Assistant professor	0.09	0.10	0.11	0.07	+
	Associate/full professor	0.14	0.14	0.20	0.13	*
	Employee	0.17	0.18	0.17	0.15	ns
	Individual/no affiliation	0.05	0.06	0.03	0.04	ns
	Other position	0.02	0.02	0.03	0.02	ns
	Position unknown	0.03	0.01	0.01	0.07	**
**Gender**	Male	0.57	0.58	0.56	0.53	ns
	Female	0.40	0.37	0.40	0.45	ns
	Gender N/A or unknown	0.04	0.05	0.04	0.02	+

Differences between columns 3 and 4 tested using Stata’s test of proportions (prtest)

^+^ = sig. at 10%

* = sig. at 5%

** = sig. at 1%.

**Table 3:** Summary statistics at the campaign level (including average creator characteristics).

	Variable	Mean	SD	Min	Max
**Affiliation**	Share educational	0.80		0	1
	Share firm	0.05		0	1
	Share other organization	0.09		0	1
	Share none/independent	0.06		0	1
**Position**	Share below PhD/MD	0.23		0	1
	Share PhD/MD	0.22		0	1
	Share Postdoc	0.05		0	1
	Share assistant professor	0.10		0	1
	Share associate/full professor	0.12		0	1
	Share employee	0.18		0	1
	Share individual/no affiliation	0.06		0	1
	Share other position	0.02		0	1
	Share position unknown	0.02		0	1
**Gender**	Share male	0.58		0	1
	Share female	0.38		0	1
	Share n/a or unknown	0.04		0	1
**Region**	US south	0.15		0	1
	US northeast	0.31		0	1
	US pacific	0.22		0	1
	US west/midwest	0.17		0	1
	Non-US	0.11		0	1
	Region unknown	0.03		0	1
**Other creator characteristics**	Creator count	1.58	1.10	1	7
**Field**	Biology	0.51		0	1
	Ecology	0.32		0	1
	Engineering	0.13		0	1
	Medicine	0.25		0	1
	Education	0.12		0	1
	Psychology	0.11		0	1
	Social Science	0.08		0	1
	Chemistry	0.05		0	1
	Other field	0.24		0	1
**Objective**	Research	0.78		0	1
	Development	0.12		0	1
	Other goal	0.10		0	1
**Budget**	Total budget	7,791	38,185	50	1,000,000
	Share creator salary	0.03	0.14	0	1
	Share other salary	0.11	0.25	0	1
	Share equipment	0.60	0.40	0	1
	Share travel	0.16	0.29	0	1
	Share other direct	0.10	0.24	0	1
	Share indirect cost	0.00	0.01	0	0.3
	Share other	0.01	0.07	0	1
**Other project characteristics**	Funding target	6,460	37,473	100	1,000,000
	Risk score	15.81	8.81	0	60.44
	Risk score simple	13.48	10.04	0	70.00
**Campaign characteristics**	Endorsement 01	0.15		0	1
	Prior papers: None	0.74		0	1
	Prior papers: 1	0.08		0	1
	Prior papers: 2	0.04		0	1
	Prior papers: 3+	0.05		0	1
	Prior papers: mentioned/link	0.08		0	1
	Video 01	0.58		0	1
	Lab notes pre closing 01	0.68		0	1
	Rewards 01	0.11		0	1
	Platform age	110	34	0	179
**Outcomes**	Funded 01	0.48		0	1
	Amount raised	6,358	98,203	0	2,641,086
	Press coverage 01	0.20		0	1

### Creator characteristics

**Affiliation**. Campaigns typically provide information on the background of the creators. If the campaign did not provide this information, we searched the internet. We hand-coded the organizational affiliations of the creators using the following categories: Educational institution (including universities, colleges, and high schools); company/firm (including startups as well as established firms); and other organization (including non-profits or government research institutes). Some creators acted without organizational affiliation, sometimes explicitly stating that they were “independent”; these are coded as “no affiliation/independent”.

**Position**. We coded creators’ position using the following categories: Student below PhD/MD level; PhD/MD student; Postdoctoral researcher; Assistant Professor; Associate/Full Professor; Employee/Affiliate (if not one of the above categories); individual (no affiliation); and other. If campaigns listed teams of individuals with clear organizational positions (e.g., a team of undergraduate students participating in an iGEM contest), we coded them accordingly. The “other” category of positions includes cases where the creators are teams of unknown composition or organizations (e.g., a foundation).

**Gender**. We coded creators’ gender primarily based on first names using the API of genderize.io. The algorithm returns the gender and a probability that a specific name-gender attribution (male or female) was correct; in case it cannot decide, the algorithm returns none. In a second step, we double-checked the accuracy of the codes and completed missing data with additional help from the profile pictures of creators or googling their name. Gender is set to “N.A./unknown” if the primary organizer is a team or an organization.

**Region**. Many campaigns include a tag indicating the primary affiliation of the creators (e.g., name of a university or company). If such an affiliation was not provided, we coded the location of the researchers based on the project description or researcher profiles. Only 5% of campaigns have more than one location and we thus focus on the primary one. Note that the coding reflects the location of the researchers, which may differ from the location where research is performed (e.g., a campaign by Duke University researchers to study the Brazilian rainforest would be coded as located at Duke). We code the following broader regions: Non-US, US-Northeast (IL, IN, OH, PA, NJ, RI, CT, MA, NH, VT, ME, NY, MI, WI), US-South (FL, MS, AL, GA, SC, NC, TN, KY, WV, VA, DC, MD, DE), US-West/Midwest (TX, LA, AR, OK, NM, AZ, NV, UT, CO, KS, MO, IA, NE, WY, ID, MT, SD, MN, ND), and US-Pacific (CA, OR, WA, AK, HI). Although it would be desirable to analyze data at the level of individual states, many states have too few cases for reliable inference.

**Creator count**. This is the count of creators listed on the campaign webpage.

### Project characteristics

**Field**. Creators indicated up to 5 field classifications on the campaign website. We coded a series of 20 dummy variables taking the value of one if a particular field was selected (see Table 3). We collapsed very small fields (fields with less than 5% of cases) into the field “Other”.

**Project objective**. We coded the substantive project objective by manually classifying projects into the following categories: Projects whose main objective is conducting scientific research; projects that focus on development (e.g. the development of devices, tools, software, and methods); and projects with other objectives (e.g., the restoration of objects or the protection of animals and ecosystems). We classified projects as research if they focused on identifying general mechanisms or empirical regularities. In many cases, creators of research projects also stated their goal to publish results in a scientific journal. This coding scheme is relatively simple and does not distinguish projects that might pursue multiple objectives [38]. However, most projects are very small with a clearly defined goal, and drawing more nuanced distinctions was not possible in a reliable way.

**Funding target**. Campaign pages originally showed only the amount raised and the percentage of the target that has been raised. We recover the target by dividing the amount raised by the percentage raised. For campaigns that raised no money, we obtained targets from updated webpages. For descriptive analyses, we report figures in U.S. Dollars. Given the skewed distribution of funding targets, we log-transform this variable for use in regression analyses.

**Budget**. Campaigns include a budget that shows the intended use of funds. Experiment.com does not provide pre-defined budget categories and we hand-coded expenses into the following categories: Salaries for organizers (individuals listed as creators on the campaign); salaries for non-organizers (e.g., students, research assistants); equipment, materials, supplies, software, and analysis services; travel (including conferences and field trips); other direct costs (e.g., compensation for patients, publications, open access fees); indirect costs (overhead); other (including budget without details). We then compute for each project the share of costs in each category.

**Risk score**. We analyze the text of the project description to measure the degree to which a project is described as risky by its creators. To do so, we use an algorithm that calculates a score for each project based on the frequency of terms typically associated with risk. More specifically, we employ the commonly used word list developed by Loughran and McDonald [44–46], which is based on the union of uncertain, weak modal, and negative words. Examples of uncertain words include *believe*, *pending*, *approximate*, *uncertain*, and *uncertainty*. Examples of negative words include *failure*, *decline*, and *difficult*. Examples of weak modal words include *could*, *might*, *nearly*, *maybe*, and *possibly*. We use two versions of the text-based risk score. Our main version is the Term Frequency-Inverse Document Frequency (TF-IDF) score, which gives more weight to words that are relatively rare in the entire corpus of documents and should thus be more informative and helpful in distinguishing projects [47]. This score also includes normalization to account for the different length of project descriptions, addressing the concern that a given word is more likely to occur in a longer text [48]. For a robustness check, we also use the simple term frequency, i.e., the count of terms from the word list. Given that project creators can choose how to pitch their project, the risk score should be interpreted as a measure of the degree to which they describe a project as risky, which may or may not correlate with the “objective” riskiness of the project.

### Campaign characteristics

**Endorsements 01**. Experiment.com offers creators the option to show endorsements by professional scientists or other individuals. We coded a dummy variable equal to one if the campaign lists at least one endorsement.

**Prior papers**. Some campaigns list prior peer reviewed publications of creators. Such publications may allow researchers to signal their accomplishment and scientific credibility. We coded a set of five categorical variables: No publications mentioned (omitted category); one specific publication listed; two specific publications listed; three or more specific publications listed. The final dummy captures if creators do not list specific publications but explicitly mention their publication record (e.g., “I have published over 100 peer reviewed articles”) or provide an explicit link to an external website that lists publications (e.g., “You can find my publication list here”).

**Video 01**. Dummy variable equal to one if the campaign includes a video that introduces the creators and/or the project.

**Lab notes pre closing 01**. Experiment.com allows creators to provide background information and campaign updates in the form of “lab notes”. We created a dummy variable equal to one if creators posted at least one lab note prior to the closing of the campaign. This variable may reflect that creators are willing to engage more actively with potential funders.

**Rewards 01**. Campaigns may offer rewards to backers for making a pledge. Examples of rewards include photographs of animals, lab visits, or T-shirts. We coded a dummy variable equal to one if a campaign offered any rewards. Although some campaigns make access to lab notes contingent on a donation, contingent lab note access is not counted as a reward in our coding.

**Platform age**. To control for the age of the platform at the time that a particular campaign is run, we compute the time difference between the closing of the focal campaign and the closing of the first campaign on the platform (May 18, 2012), measured in weeks. All regressions control for platform age and age squared.

#### Outcomes

**Funded 01**. Dummy variable equal to one if the campaign raised at least 100% of its target.

**Amount raised**. Amount raised by the campaign, regardless of whether the target was reached. Note that campaigns may raise more than their target. For descriptive analyses, we report figures in U.S. Dollars. Given the skewed distribution of this measure, we log-transform this variable for regression analyses.

**Press coverage 01**. Some campaigns list press coverage of the campaign itself or of the creators’ larger research programs. Such coverage may include national media such as the New York Times and Discover Magazine, local newspapers, radio shows, or coverage by third party websites. Given the relatively small numbers, we use a simple dummy variable equal to one if the campaign lists at least one press item. We use this variable for two purposes. First, we include this variable in regressions of financial funding outcomes because press coverage listed on the campaign website may serve as a quality signal for potential backers. The buzz created by press coverage may also attract new backers to the campaign. Second, science writers and reporters constitute a somewhat different audience than the regular crowd, and may be more likely to have scientific training or relevant experience [49]. As such, we also use this variable as dependent variable in exploratory analyses to proxy for success in attracting attention from a more professional audience.

## Results

### Selected descriptive insights

**Fig 1:**
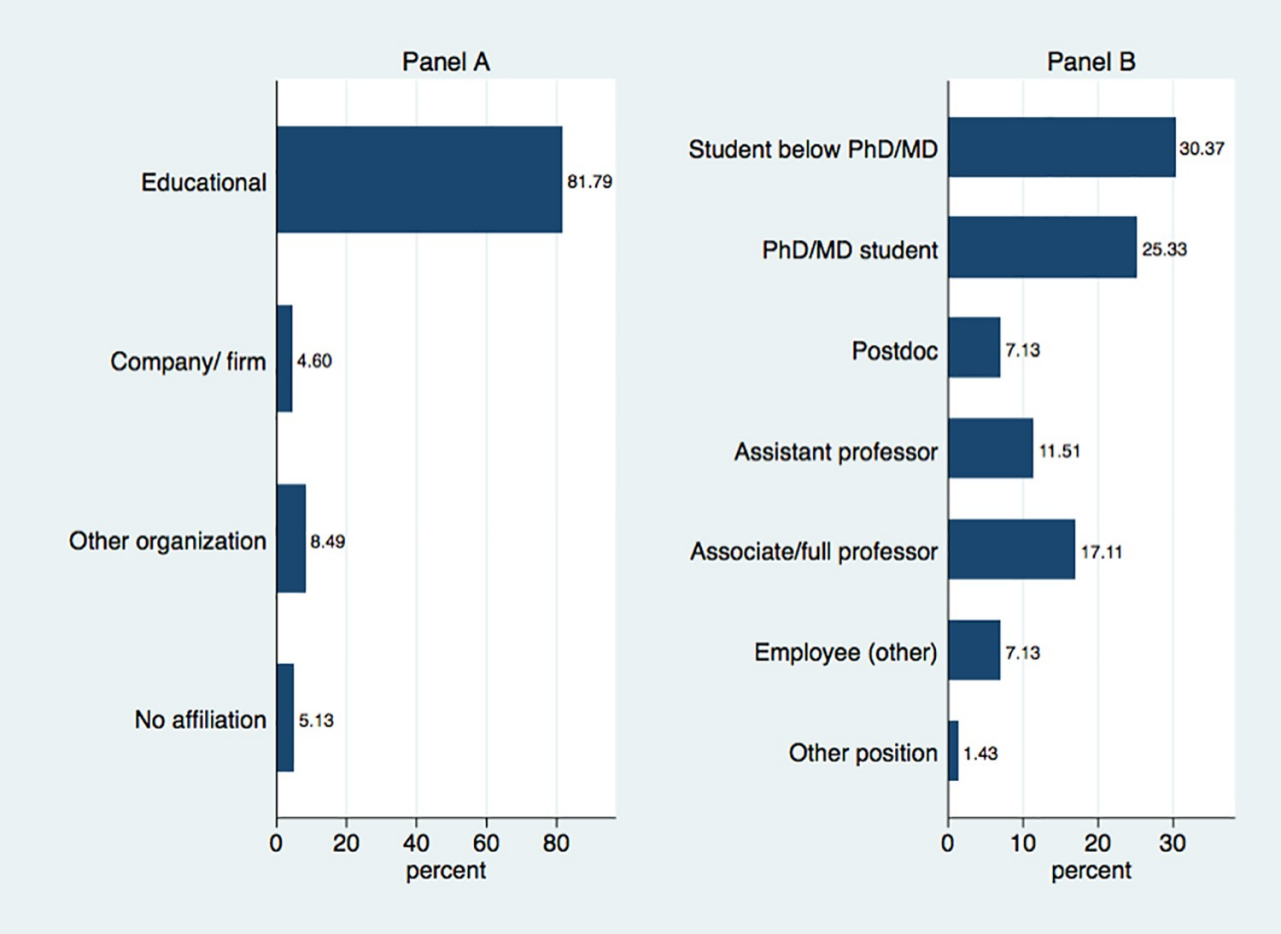
Characteristics of project creators. (A) Affiliation of all project creators (N = 1,131). (B) Position of creators who are affiliated with an educational institution (N = 912). Excludes cases with missing data.

**Creator characteristics**. We first examine key characteristics of the creators who started crowdfunding campaigns. Panel A in Fig 1 shows the affiliation of the creators. Over 80% are affiliated with educational institutions (e.g., universities and colleges), about 5% are affiliated with firms, and 8% with other organizations such as foundations, museums, non-profits, or research institutes. Roughly 5% of creators are un-affiliated, sometimes explicitly calling themselves “independent researcher”. The preponderance of campaigns involves creators from just one type of affiliation. In particular, of all the campaigns with at least one creator from an educational institution, only 2% also have a creator affiliated with a firm, and only 6% also have a creator affiliated with an “other” organization (e.g., nonprofit, research institute).

We further distinguish creators affiliated with educational institutions by their position (Fig 1, Panel B). We find that a large share of these creators are students, including over 30% undergraduate or master’s students and 25% PhD or MD students. Roughly 7% are postdocs, 12% are assistant professors, and 17% are associate or full professors.

With respect to gender, 57% of all campaign creators are male and 40% are female. In the remaining cases, gender could not be determined or did not apply because an organization, not a person, was listed as the creator.

As noted earlier, 68% of campaigns were started by a single creator while 32% were started by teams. Table 2 shows creator characteristics separately for all creators (column 1), and for only one creator per campaign, taking the first-listed creator in case of teams (column 2). For team-based projects, we further show creators’ characteristics separately for the investigator listed first in the team (column 3), and for all other team members who were not listed first (column 4). First and non-first listed individuals in teams are quite similar in terms of affiliation, reflecting the low rate of cross-affiliation collaborations. However, first listed creators in teams are somewhat more senior (significantly less likely to be students below PhD/MD level and significantly more likely to be associate/full professors).

The vast majority of creators on the platform Experiment.com are located in the U.S. (89%) and 11% are located in other countries. The US-based creators were distributed across all regions, including northeastern states (31%), southern states (15%), states bordering the Pacific (22%), and states in the west/midwest (17%). We compared the geographic distribution of funded US-based campaigns to the distribution of awards by NIH and NSF over a comparable time period (2012–2015). S1 Fig shows that Experiment.com has a somewhat larger share of successful projects in the Pacific region (32%) and a smaller share in the northeast (34%) compared to NSF/NIH, likely because Experiment.com was started on the West Coast. Moreover, Experiment.com funding volume is heavily concentrated in the Pacific region (78%), which is partly due to an extremely successful outlier project located in California (see below). While the specific patterns are unlikely to generalize, two observations may be more general. First, at least in the first years of their operation, crowdfunding platforms may be more localized than traditional funding sources, serving primarily their home regions [26]. Second, while government grants tend to be of similar sizes [50], amounts raised in crowdfunding can vary quite dramatically. As such, the regional distribution of amounts raised may differ quite substantially from the regional distribution of the number of successful campaigns.

**Project characteristics**. After providing insights on campaign creators, we examine more closely what kinds of projects these creators propose. First, the most frequently listed field classifications are (in descending order of frequency): Biology, Ecology, Medicine, Engineering, Education, Psychology, Social Sciences, and Chemistry (Table 3).

In terms of their substantive objectives, roughly 78% of projects aim at the scientific investigation of a topic (e.g. the impact of climate change on oak trees, the use of computer games to develop team skills in autistic children, the testing of a drug against kidney cancer), and 12% aim at the development of devices, tools, software, or methods. The remaining 10% have other types of objectives, such as the restoration of objects (e.g. dinosaur skeletons) or the protection of animals and ecosystems.

As a proxy for project size, we examine the amount of funding creators seek to raise. Funding targets ranged from $100 to over $100,000. One extreme project had a target of $1 million to find a cure for the rare Batten disease [51]. The average project target was $6,460, the median $3,500. Thus, while some campaigns reach the scale of traditional funding requests, most seek to raise small amounts. One possible explanation is that crowdfunding is used for pilot studies that are intended to lead to larger follow-on projects. Although an explicit framing of the study as a pilot was not common, it occurred in several instances. For example, one campaign stated, “It is almost impossible to achieve funding without substantial preliminary data. This fundraiser will help fund this initial experiment and provide data for future grant proposals.” [52]

Campaigns also include a budget, allowing us to explore for what kinds of expenses creators seek to raise funds. The average campaign requested the majority of funds for materials, equipment and services (60%), followed by travel (16%) and salaries for personnel other than the creators (e.g. research assistants) (11%). Compensation or salary for creators constituted only 3% of the average budget.

### Predictors of fundraising success

Creators receive pledged funds only if the campaign achieves the pre-defined target at the closure date. The success rate in our sample was 48%, higher than the success rate of projects on the general-purpose platform Kickstarter (36%) [42] and considerably higher than the success rates at NSF (23% in competitive grants in 2014) and NIH (16% for new research applications in 2015) [53, 54]. Conditional upon funding success, projects raised a total of $4.37 million, distributed in a range from $110 to an extreme of over $2.6 million for the Batten disease project, with an average of $12,652 and median of $3,105. We now turn to the question how funding success is related to characteristics of the creators, the projects, and the campaigns.

**Fig 2:**
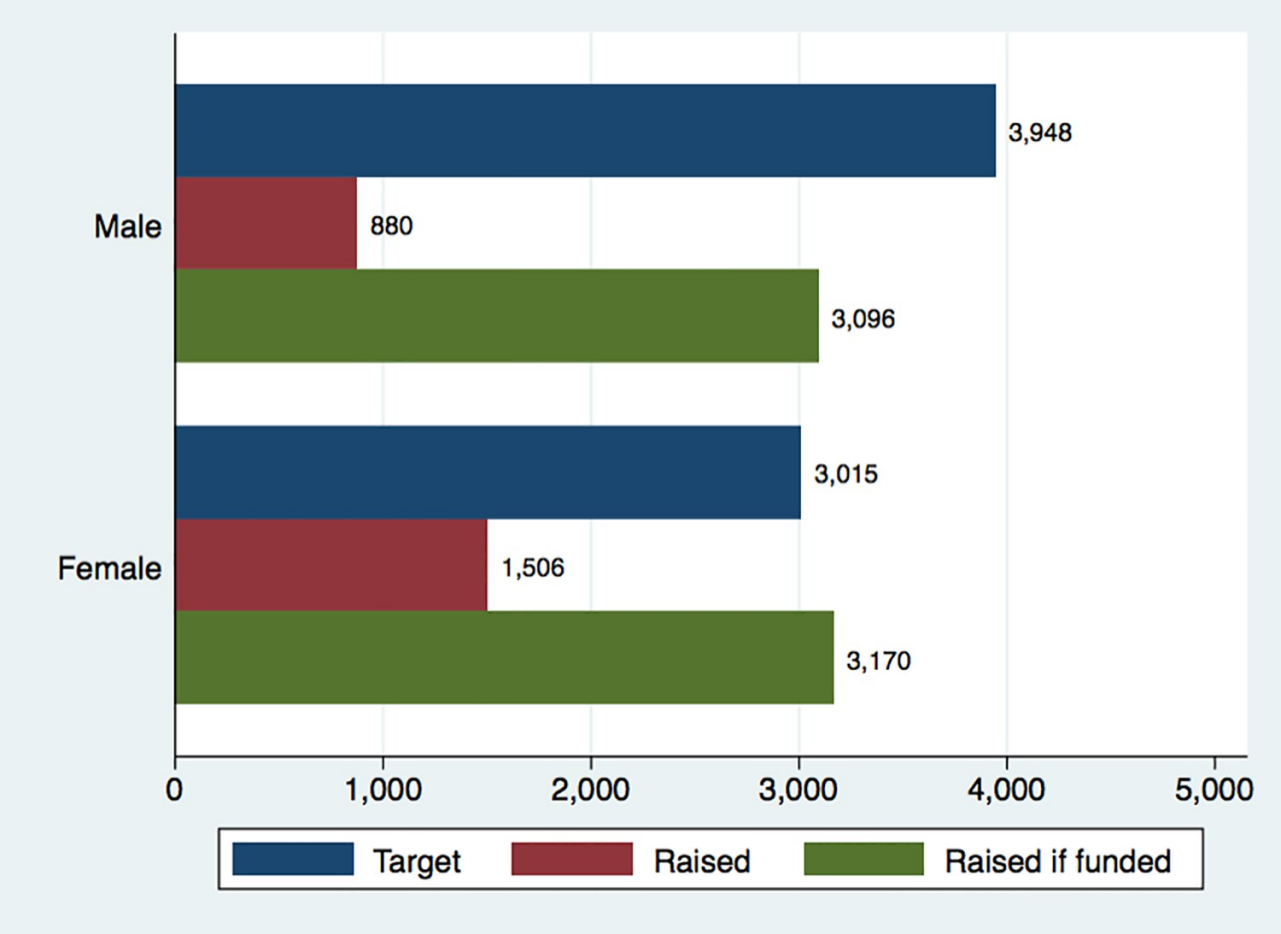
Median targets, amounts raised, and amounts raised conditional upon funding success, by gender of first author (N = 691, in USD).

**Fig 3:**
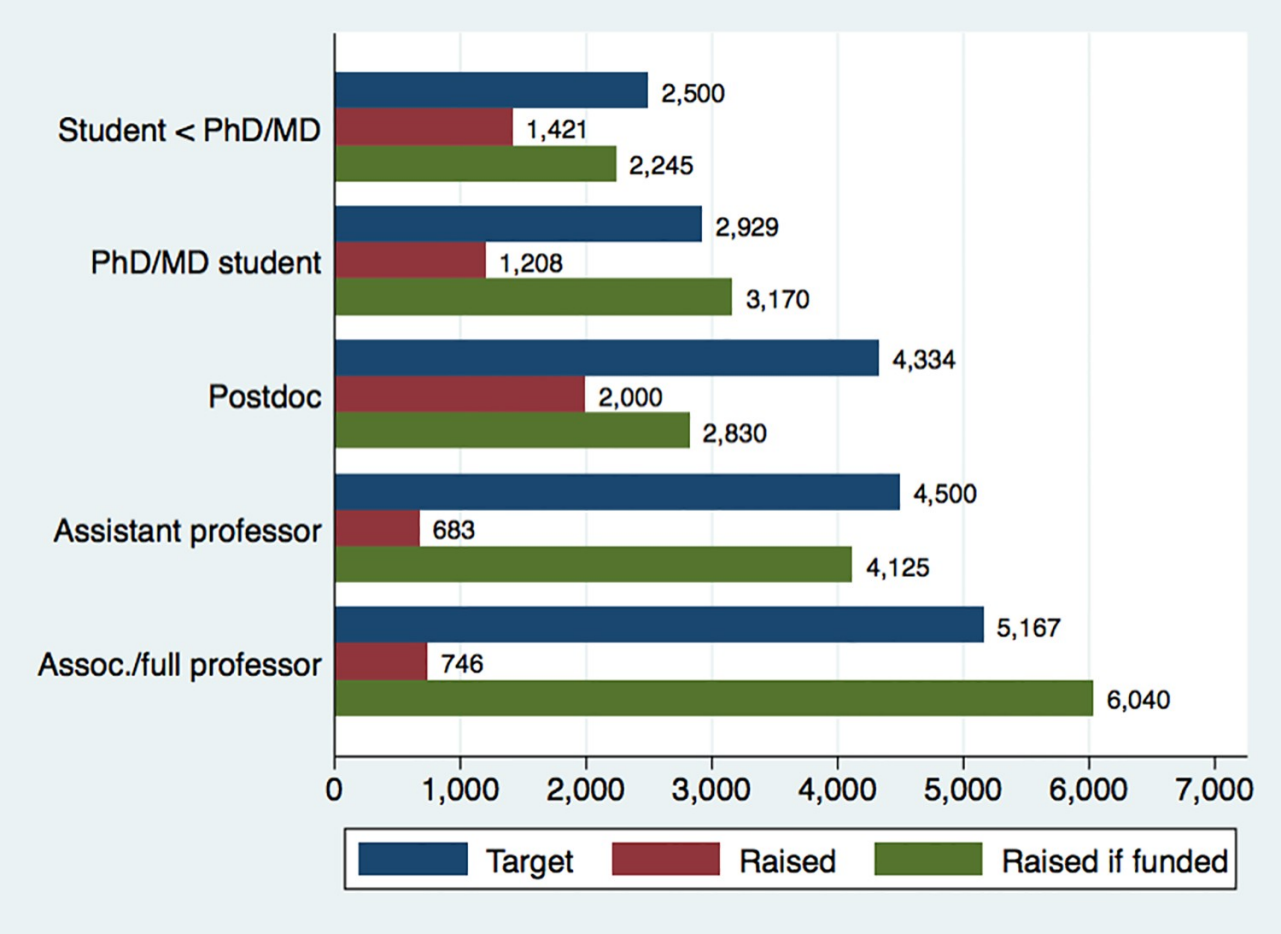
Median targets, amounts raised, and amounts raised conditional upon funding success, by position of first author (educational institutions only, other positions omitted) (N = 533, in USD).

Figs 2 and 3 illustrate differences in funding targets as well as fundraising success for two particularly relevant creator characteristics: gender and position. Fig 2 shows that men have larger funding targets than women (median of $3.948 vs. $3.015), consistent with observed gender differences on Kickstarter [55]. However, men receive a significantly lower volume of pledges ($880 vs. $1.506), resulting in lower rates of success (43% versus 57%). Conditional upon reaching the funding target, amounts raised by men and women are very similar ($3.096 versus $3.170). Fig 3 shows patterns by scientists’ position: Students and postdocs tend to have lower targets but receive a higher volume of pledges than senior scientists, resulting in higher rates of success (e.g., 61% for students below PhD/MD versus 33% for associate/full professors). Although intriguing, these patterns are difficult to interpret because they do not account for potentially confounding factors such as differences across fields or geographic regions. As such, we now examine the predictors of fundraising success more systematically using regression analysis.

**Regression analysis**. We examine predictors of fundraising success using regression models that control for factors such as scientific field, geographic region, or age of the platform (Table 4). For the 32% of campaigns created by a team, our main analysis focuses on the characteristics of the *first* listed creator. The rationale is that in the sciences, first listed authors are typically those who “own” the project and make the largest substantive contributions [56]; Experiment.com leadership confirmed in discussions that this is the case also on the platform. Campaign descriptions typically provide more information about first authors than non-first authors, providing further support for the notion that these individuals are driving the project. Robustness checks using team averages of individual characteristics (e.g., the share of female team members) rather than the characteristics of the primary creator show very similar results (reported in S2 Table).

**Table 4:** Funding outcomes.

	Funded 01				(Ln) amount raised			
	1	2	3	4	5	6	7	8
	logit	logit	logit	logit	OLS	OLS	OLS	OLS
Position: Below PhD/MD		4.034**	2.848**	3.094**		0.026	0.629*	0.569*
		[1.282]	[0.956]	[1.176]		[0.287]	[0.279]	[0.264]
Position: PhD/MD		2.379**	1.826+	1.523		0.170	0.598*	0.379
		[0.726]	[0.588]	[0.530]		[0.268]	[0.255]	[0.238]
Position: Postdoc		3.697**	3.281**	2.099+		0.670*	0.938**	0.413
		[1.539]	[1.400]	[0.924]		[0.299]	[0.292]	[0.279]
Position: Assistant professor		1.158	0.982	0.818		-0.209	0.055	-0.153
		[0.417]	[0.363]	[0.332]		[0.334]	[0.339]	[0.302]
Position: Associate/full professor		omitted	omitted	omitted		omitted	omitted	omitted
Position: Employee		1.226	0.878	0.845		-0.539	-0.037	-0.016
		[0.540]	[0.408]	[0.413]		[0.398]	[0.406]	[0.371]
Position: Individual/no affiliation		2.498*	1.902	1.741		-0.286	0.270	0.109
		[1.073]	[0.820]	[0.920]		[0.395]	[0.429]	[0.406]
Position: Other position		2.283	2.404	3.055+		0.207	0.250	0.211
		[1.503]	[1.554]	[2.029]		[0.725]	[0.678]	[0.664]
Affiliation: Educational institution		omitted	omitted	omitted		omitted	omitted	omitted
Affiliation: Firm		1.102	1.574	1.833		0.998*	0.615	0.553
		[0.587]	[0.892]	[1.097]		[0.434]	[0.440]	[0.390]
Affiliation: Other organization		1.996	2.288+	1.971		1.061**	0.829*	0.556
		[0.881]	[1.039]	[0.961]		[0.380]	[0.379]	[0.344]
Gender: Female		1.518*	1.551*	1.540*		0.407**	0.394**	0.308*
		[0.277]	[0.290]	[0.310]		[0.152]	[0.145]	[0.131]
Gender: N/A or unknown		0.681	0.745	0.693		0.209	0.053	0.028
		[0.335]	[0.366]	[0.349]		[0.430]	[0.386]	[0.342]
(Ln) target			0.650**	0.500**			0.640**	0.495**
			[0.065]	[0.059]			[0.081]	[0.079]
Objective: Research			omitted	omitted			omitted	omitted
Objective: Development			0.761	0.857			-0.357	-0.244
			[0.234]	[0.275]			[0.264]	[0.239]
Objective: Other			1.156	1.222			-0.147	-0.094
			[0.386]	[0.434]			[0.285]	[0.272]
Risk score			0.988	0.994			-0.009	-0.002
			[0.009]	[0.011]			[0.007]	[0.007]
Prior pubs: 1 listed				1.558				0.462+
				[0.591]				[0.240]
Prior pubs: 2 listed				1.059				0.498*
				[0.540]				[0.245]
Prior pubs: 3+ listed				0.860				-0.415
				[0.339]				[0.315]
Prior pubs mentioned/linked				0.496*				-0.086
				[0.172]				[0.226]
Endorsements 01				2.699**				0.616**
				[0.755]				[0.144]
Video 01				1.701*				0.457**
				[0.352]				[0.145]
Lab notes pre closing 01				3.769**				1.381**
				[0.828]				[0.162]
Reward 01				2.299*				0.539**
				[0.847]				[0.178]
Press coverage 01				1.609+				0.016
				[0.397]				[0.164]
Creator count	1.137+	1.176*	1.219*	1.150	0.241**	0.218**	0.196**	0.131*
	[0.087]	[0.096]	[0.105]	[0.104]	[0.069]	[0.073]	[0.070]	[0.061]
Region fixed effects	incl.	incl.	incl.	incl.	incl.	incl.	incl.	incl.
Field fixed effects	incl.	incl.	incl.	incl.	incl.	incl.	incl.	incl.
Platform age	incl.	incl.	incl.	incl.	incl.	incl.	incl.	incl.
Constant	2.793	1.086	57.907**	220.852**	7.783**	7.582**	1.717+	1.729*
	[2.212]	[0.866]	[73.802]	[330.834]	[0.600]	[0.635]	[0.908]	[0.838]
Observations	721	721	721	721	721	721	721	721
df	17	28	32	41	17	28	32	41
Pseudo R-squared	0.125	0.165	0.187	0.279				
R-squared					0.143	0.174	0.255	0.397

Robust standard errors in brackets. Odds ratios reported for logits (values <1 indicate a negative relationship).

^+^ = sig. at 10%,

* = sig. at 5%,

** = sig. at 1%.

We use two different variables to capture fundraising outcomes: The first is the dummy variable indicating whether a campaign achieved its target (Table 4, Models 1–4). These models are estimated using logistic regression, and we report odds ratios for ease of interpretation (odds ratios greater than one indicate a positive relationship, odds ratios smaller than one indicate a negative relationship). The second dependent variable is the continuous measure of pledges received regardless of whether the funding target was achieved (Models 5–8). These regressions are estimated using OLS. All regressions use Huber-White “robust” standard errors to address potential heteroscedasticity.

For each of the two outcome variables, we estimate four models. The first model includes just control variables. The second also includes characteristics of the primary campaign creator. The third additionally includes characteristics of the project. The fourth model additionally includes characteristics of the campaign. For both outcome variables, each of these steps meaningfully increases model fit, with the full models more than doubling the (Pseudo)R^2^ of the baseline models. The regressions allow us to examine how success rates are related to characteristics of creators, characteristics of the projects, and to how campaigns are implemented. The stepwise approach also provides some insights into the degree to which differences in the baseline success rates of different types of creators (Models 2 and 6) may be explained by differences in project or campaign characteristics [57].

**Funding success and creator characteristics**. Model 2 in Table 4 shows that that students and postdocs are more likely to reach their funding targets than associate and full professors. These differences are significantly reduced (Chi^2^(3) = 14.49, p<0.01) but remain significant when we account for the fact that their targets tend to be lower, and thus easier to achieve (Model 3). Students and postdocs also receive a greater volume of pledges (Table 4, Model 6), especially when we account for differences in funding targets (Model 7). These results are correlational and we can only speculate about the underlying mechanisms. One possible interpretation is that the crowd favors junior scientists, e.g., because backers consider perceived need in addition to scientific merit, or derive utility from supporting the education and professional development of students and postdocs. Alternative explanations are that junior scientists may have more creative project ideas [58], invest more effort in campaigns, or are better able to engage with non-expert audiences. Finally, associate and full professors that resort to crowdfunding may propose projects that are “adversely selected”, e.g., lower quality ideas that were rejected by traditional funding sources.

We find no systematic differences in funding success between creators affiliated with educational institutions versus any other type of organizations. Since measures of positions are correlated with affiliation types, we also re-estimate regressions with measures of positions only; the substantive results are unchanged (S3 Table, Models 1–4).

Consistent with prior research [55], we find significant gender differences in crowdfunding success: Women have higher odds of reaching their funding goal than men (Table 4, Model 2) and also raise significantly more money (Model 6). The gender dummy changes little when project and campaign characteristics are included (Models 3, 4, 7, and 8). Women’s significantly higher success rates in crowdfunding contrast with similar or slightly lower odds of success than men when competing for grants from government agencies such as NIH or NSF [59, 60]. Future research is needed to explore potential drivers of the observed gender differences in crowdfunding success and better data on the gender of project backers would be particularly valuable [21].

**Funding success and project characteristics**. We now turn more explicitly to the characteristics of the project for which funding is sought (Table 4, Models 3 and 7). We find no significant differences in funding success between projects pursuing research versus development objectives. Projects with larger funding targets are less likely to get funded, consistent with prior evidence from general purpose crowdfunding [2]. At the same time, campaigns with higher targets receive a higher volume of pledges, highlighting the challenge of setting funding targets that are achievable but also result in meaningful resources when achieved.

Our text-based measure of the degree to which creators convey risks of a project has no relationship with funding success or the amount of money raised (Table 4, Models 3 and 7). Robustness checks using an alternative simpler risk measure (term frequency) give the same result (S3 Table, Models 5–8). Although our analysis cannot address the question whether the crowd funds more or less risky projects than traditional funding agencies [61], it suggests that backers on dedicated platforms for funding scientific research may pay little attention to how extensively creators discuss risks.

**Funding success and campaign characteristics**. The data show several relationships between funding success and features of the campaign itself (Table 4, Models 4 and 8). First, a potential challenge in crowdfunding research is that backers–especially those without a background in science–may find it difficult to assess the scientific merit of projects or creators. Campaigns can address this challenge by using various quality signals [1]. For example, Experiment.com offers campaign creators the option to include endorsements by professional scientists or other individuals. We find that campaigns with endorsements (15% of all campaigns) have significantly higher odds of success and raise more funds than campaigns without endorsement. Another potential quality signal is the listing of prior publications that are (co-)authored by the creators. We find that listing specific publications has no significant relationship with the likelihood of getting funded (Model 4), although it is associated with somewhat higher amounts raised (Model 8). Interestingly, just mentioning the presence of prior publications or linking to a publication list is associated with lower likelihood of funding. Anecdotally, we noticed that when campaigns list specific publications, these tend to be clearly related to the project content and are sometimes integrated in the project narrative. In contrast, mentioning the presence of publications (“I have published over 100 peer reviewed articles”) or linking to publication lists is more common in sections on creator background and may be meant primarily to demonstrate scientific accomplishment. Although the observed relationships may reflect the crowd’s reaction to publications per se, they may also reflect otherwise unobserved heterogeneity in the nature of projects or project creators. Either way, the lack of a positive relationship between prior publications and funding is interesting given the important role that prior publications play in traditional grant applications [36].

Second, Experiment.com allows creators to provide background information and campaign updates in the form of “lab notes”. Campaigns that posted lab notes prior to closure (68% of all campaigns) have significantly higher odds of success and receive a higher volume of pledges than projects without lab notes. Campaigns featuring a video presentation (58%) are also more likely to succeed, consistent with other crowdfunding contexts [2]. These results reinforce the notion that effort in designing campaigns as well as reaching out and interacting with the crowd can be an important predictor of funding success [19, 62].

Finally, prior studies argue that research projects find it difficult to offer the kinds of tangible rewards that are often central to crowdfunding campaigns on platforms such as Kickstarter [17]. Consistent with this concern, most projects in our sample do not offer any rewards. However, 11% of projects offer–sometimes quite creative–rewards such as visits to the research lab, acknowledgement on future publications, photographs of animals observed, or the naming of a shark. Projects offering a reward have a higher likelihood of achieving their target and also raise significantly more funds.

The prior analyses showed that one of the primary predictors of funding success is the funding target (e.g., Table 4, Models 3 and 7). To better understand how the funding target differs across different types of creators and how it is correlated with other characteristics of the project and the campaign, we estimate regressions with (Ln) funding target as dependent variable (Table 5, Models 1–4). These regressions show that the gender differences in funding targets observed in Fig 2 are not significant when control variables are included. However, they reinforce the patterns seen in Fig 3, i.e., significantly higher targets among senior scientists. This may reflect that senior scientists work on “bigger” projects, but also that they are more ambitious (and potentially overconfident) with respect to the amount of funding sought.

**Table 5:** Funding targets and press coverage.

	(Ln) target				Press coverage 01			
	1	2	3	4	5	6	7	8
	OLS	OLS	OLS	OLS	logit	logit	logit	logit
Position: Below PhD/MD		-0.894**	-0.878**	-0.780**		0.308**	0.472*	0.572
		[0.115]	[0.117]	[0.116]		[0.108]	[0.176]	[0.230]
Position: PhD/MD		-0.664**	-0.665**	-0.649**		0.548+	0.692	0.694
		[0.115]	[0.114]	[0.109]		[0.180]	[0.240]	[0.260]
Position: Postdoc		-0.414*	-0.414*	-0.525**		1.032	1.222	1.048
		[0.161]	[0.162]	[0.161]		[0.442]	[0.520]	[0.480]
Position: Assistant professor		-0.389**	-0.379**	-0.399**		0.897	1.044	1.073
		[0.132]	[0.132]	[0.132]		[0.319]	[0.389]	[0.434]
Position: Associate/full professor		omitted	omitted	omitted		omitted	omitted	omitted
Position: Employee		-0.791**	-0.786**	-0.713**		0.552	0.730	0.681
		[0.185]	[0.185]	[0.178]		[0.277]	[0.361]	[0.349]
Position: Individual/no affiliation		-0.755**	-0.712**	-0.649**		0.357+	0.534	0.648
		[0.184]	[0.184]	[0.187]		[0.194]	[0.305]	[0.384]
Position: Other position		-0.006	-0.006	0.061		1.341	1.555	1.197
		[0.369]	[0.363]	[0.364]		[1.233]	[1.523]	[1.307]
Affiliation: Educational institution		omitted	omitted	omitted		omitted	omitted	omitted
Affiliation: Firm		0.723**	0.743**	0.652**		0.743	0.633	0.730
		[0.226]	[0.228]	[0.211]		[0.506]	[0.407]	[0.485]
Affiliation: Other organization		0.442*	0.489*	0.411*		0.948	0.901	1.050
		[0.203]	[0.208]	[0.202]		[0.497]	[0.480]	[0.570]
Gender: Female		0.005	-0.002	-0.002		1.271	1.277	1.339
		[0.075]	[0.076]	[0.074]		[0.270]	[0.274]	[0.295]
Gender: N/A or unknown		0.259	0.271	0.255		0.501	0.386	0.492
		[0.190]	[0.190]	[0.192]		[0.389]	[0.359]	[0.425]
(Ln) target							1.564**	1.400**
							[0.186]	[0.167]
Objective: Research			omitted	omitted			omitted	omitted
Objective: Development			-0.099	-0.068			0.624	0.738
			[0.124]	[0.125]			[0.234]	[0.290]
Objective: Other			-0.179	-0.138			0.580	0.619
			[0.134]	[0.137]			[0.239]	[0.264]
Risk score			-0.003	-0.002			1.000	1.003
			[0.004]	[0.004]			[0.012]	[0.012]
Prior pubs: 1 listed				0.111				3.786**
				[0.127]				[1.282]
Prior pubs: 2 listed				0.029				3.745**
				[0.189]				[1.669]
Prior pubs: 3+ listed				0.123				2.437*
				[0.148]				[1.073]
Prior pubs mentioned/linked				0.309*				1.079
				[0.130]				[0.406]
Endorsements 01				0.154				1.308
				[0.104]				[0.368]
Video 01				0.297**				1.821*
				[0.071]				[0.451]
Lab notes pre closing 01				0.111				1.520+
				[0.075]				[0.382]
Reward 01				-0.002				1.178
				[0.105]				[0.367]
Press coverage 01				0.212*				
				[0.082]				
Creator count	0.090**	0.051	0.056+	0.044	1.117	1.046	1.047	1.027
	[0.033]	[0.032]	[0.032]	[0.033]	[0.092]	[0.091]	[0.097]	[0.096]
Region fixed effects	incl.	incl.	incl.	incl.	incl.	incl.	incl.	incl.
Field fixed effects	incl.	incl.	incl.	incl.	incl.	incl.	incl.	incl.
Platform age	incl.	incl.	incl.	incl.	incl.	incl.	incl.	incl.
Constant	8.652**	9.224**	9.268**	8.939**	0.004**	0.011**	0.000**	0.000**
	[0.320]	[0.322]	[0.324]	[0.339]	[0.005]	[0.012]	[0.000]	[0.000]
Observations	721	721	721	721	721	721	721	721
df	17	28	31	40	17	28	32	40
Pseudo R-squared					0.0591	0.0888	0.114	0.168
R-squared	0.089	0.188	0.191	0.245				

Robust standard errors in brackets. Odds ratios reported for logits (values <1 indicate a negative relationship).

^+^ = sig. at 10%,

* = sig. at 5%,

** = sig. at 1%.

### Attention from the press

Our findings that students and postdocs tend to be more successful and that prior publications have little relationship with funding success suggest that the crowd may apply different criteria than traditional funders when deciding which projects to support. Unfortunately, we cannot test this conjecture explicitly since we lack expert evaluations of the projects in our sample. However, we can broaden our analysis by exploring the judgments of another audience, namely writers who cover project creators and their crowdfunding campaigns in the press. The predictors of press coverage may differ from the predictors of fundraising success for several reasons. First, science writers and reporters may be more likely than the general public to have advanced scientific training [49] and subscribe more strongly to traditional criteria when evaluating the importance and promise of research. Second, many of the backers in crowdfunding are friends and family [63] and our interviews with Experiment.com leadership and creators suggests that the share of friends and family among backers averages more than 50%. Science writers are less likely to be socially connected to creators and may thus apply different and more “objective” criteria than backers. Finally, the decision to cover a campaign is different from the decision to fund it and, as such, writers may emphasize different criteria than potential backers. Exploring how characteristics of creators, projects, or campaigns are associated with the likelihood of press coverage is interesting per se, but is also relevant because press coverage may in turn influence fundraising success.

Models 5–8 in Table 5 show results from logistic regressions using press coverage as the dependent variable. We find several significant relationships, including intriguing differences between the factors that predict funding success versus attention from the press. First, students are more likely to be funded than senior scientists, but they are less likely to receive press coverage. Second, lab notes and rewards are associated with significantly higher funding success but they have no significant relationship with press attention. Third, listing specific prior publications–demonstrating relevant prior work–has no relationship with funding success but does have a strong positive relationship with press coverage. Once publications are included, students’ lower odds of press coverage become insignificant, partly reflecting that students are less likely to list publications than senior scientists (see correlations in S1 Table). These relationships do not necessarily mean that the press pays attention to seniority and listed publications per se; they may also reflect differences in underlying project characteristics such that those projects that are initiated by senior scientists and associated with a larger research program are judged by the press to be of broader interest or potential impact.

Although science writers’ preferences are likely not representative of professional scientists or of decision makers at traditional funding agencies, our results provide tentative evidence that the crowd judges research projects differently from other kinds of evaluators. Similar evidence has been found in recent work comparing crowd and expert evaluations in the context of the arts [64]. Further exploration of such differences may yield important insights for project creators as well as platform designers. Scholars studying differences in the decision criteria used by the crowd and other audiences may benefit from complementing commonly used theories of information asymmetries [1, 34] with models that highlight social factors such as gift exchange, social networks, or identity.

Before we conclude, we note that all regressions in this paper should be interpreted as correlational in nature. Thus, significant coefficients on independent variables do not necessarily imply a causal effect of these variables on funding outcomes or on press attention. Causal interpretation is difficult because independent variables may proxy for otherwise unobserved factors such as the nature of research, researchers’ skills and ability, or unobserved outreach activities by creators. The statistically and economically significant relationships observed in our data suggest fruitful avenues for future research examining why exactly certain characteristics of creators, projects, and campaigns are associated with higher fundraising success or a higher likelihood of press coverage.

## Discussion

Crowdfunding for scientific research is still in its early stages, but the considerable number of funded projects suggests that it can provide important financial benefits. Moreover, crowdfunding seems to differ in important ways from traditional funding mechanisms such as grants from government agencies: Success rates are comparatively high, junior scientists tend to be more successful than senior scientists, and female investigators are more likely to be funded than male investigators. Furthermore, we find no evidence that projects described as riskier have a lower likelihood of being funded and creators’ prior publications appear to matter little for funding success. Fundraising is also faster than in the traditional grant-based system. At the same time, the amounts raised with crowdfunding tend to be much smaller and funds are used primarily for materials and travel rather than salaries or tuition.

Our results support the view that crowdfunding of scientific research broadens access to resources for groups that have been excluded or disadvantaged in traditional funding systems, similar to what has been shown in crowdfunding of business initiatives [27]. However, the amount of resources raised–at the level of individual projects but also the platform as a whole–is presently too small for crowdfunding to serve as a substitute for traditional funding mechanisms. As such, crowdfunding for research may best be seen as a complement to such traditional sources. In particular, crowdfunding appears to be particularly useful for students and postdocs who do not have the track record required by most traditional funding agencies, and it may be suitable for smaller projects and early-stage studies without preliminary evidence. In these areas, even a relatively small grant can enable a project to proceed and may also make a long-term difference by allowing researchers to increase their chances of subsequent funding in the traditional system [27].

Despite these benefits, there are also potential concerns and need for public discussion. First, the crowd may fund projects that are in legal (or political) grey zones, and there may be different views regarding the desirability and value of such research. For example, some creators in our sample noted that traditional funding sources would not support projects on gun culture [65], on the impact of the legalization of marijuana, or on the development of molecules that can lead to mutations in humans. Second, although Experiment.com requires that academic researchers proposing research involving human or vertebrate subjects obtain approval from Institutional Review Boards (IRB) or Institutional Animal Care and Use Committees (IACUC), it is not clear whether all creators–especially those outside academia–understand and follow guidelines for ethical and responsible research [6]. Another concern is that crowdfunding sidesteps traditional peer review and the crowd may support projects with low scientific merit [66]. Of course, backers may deliberately ignore some of the selection criteria used by traditional funding agencies (such as prior publications) and may also support campaigns for reasons other than scientific merit, e.g., to help a passionate student or enable a “fun” project. Nevertheless, platforms should require that projects provide enough information to allow potential backers to make informed decisions. Finally, studies on rewards-based crowdfunding suggest that many projects fail to deliver promised products or deliver with significant delay [2]. While such problems may simply reflect risky work or insufficient creator capabilities, they also raise concerns about dishonesty and fraud. Although this issue needs further research using data on scientific outcomes of projects, our discussions with Experiment.com leaders suggest that an interesting protection mechanism may be at work: As noted earlier, campaigns are unlikely to succeed without significant initial support from friends and family but creators with malicious intent would be unlikely to activate such support.

To raise meaningful amounts of funding, campaign creators need to reach beyond family and friends to engage broader audiences [62, 63]. Yet, reaching a broad audience requires significant effort, e.g., to develop videos, write engaging lab notes, or respond to backers’ comments and suggestions. Some creators may come to realize that the relatively small amount of money that can be raised is not worth these costs in terms of time and effort [67]. Indeed, our finding that crowdfunding is used especially by junior scientists may reflect that these scientists have lower opportunity costs of time than senior researchers, while also having less access to traditional funding sources. Junior scientists may also feel more comfortable using social media, an important component of many crowdfunding campaigns [19, 62]. More generally, the rise of crowdfunding is likely to increase the value of skills in communicating research and interacting with “citizen” audiences [61]. Such skills may be particularly important for researchers who work on topics whose value is not immediately apparent to the general public. In addition to dedicated programs such as the recently launched Lab for Open Innovation in Science [68], educators and PhD advisors should consider how they can help students develop communication skills as part of the regular research training. Indeed, running campaigns on a platform such as Experiment.com may be a useful learning tool.

Although our analysis focused on financial resources, crowdfunding may also provide several non-financial benefits. Creators can receive feedback on their research, achieve greater visibility, and see that others really care about their work [62]. Moreover, backers may continue to support projects in other ways, e.g., by offering access to infrastructure and research sites. The public might benefit from crowdfunding by gaining direct insights into the research process (e.g., via lab notes), participating in the allocation of resources for research, and by feeling empowered to contribute to the progress of science [61, 62]. Future research is needed to quantify these non-financial benefits and to develop tools that increase scientists’ ability to achieve the financial as well as non-financial objectives of their crowdfunding campaigns.

This discussion of non-financial benefits of crowdfunding highlights potential ties to another recent development—namely “crowd science” or “citizen science” projects, where scientists ask the public not for financial resources but for inputs in the form of time and knowledge [9, 69]. We suggest that future research could benefit from considering similarities (and differences) between these mechanisms for involving the public in different aspects of the scientific research process. Some of the tools and best practices developed in the context of citizen science may prove useful also in crowdfunding efforts, while findings from crowdfunding research may help citizen science projects to understand the dynamics of project participation and to increase participant engagement [2, 9, 70]. Indeed, future research could explore whether individuals who are willing to support a project with money are also more likely to support projects with time or other contributions, how projects can successfully ask for both financial and non-financial contributions, and whether platforms can benefit from co-hosting crowdfunding and citizen science projects.

Even though our focus is on understanding crowdfunding as a mechanism to raise resources for scientific research, our findings also contribute to the broader crowdfunding literature. First, some of our results corroborate prior findings in a new empirical context, suggesting broader generalizability of those prior findings. Among others, we confirm that women are not at a disadvantage but tend to be more successful than men in raising crowdfunding [21–23]. Using a rich set of measures on projects and campaigns, we also find that this advantage holds even when we account for campaign targets or characteristics of the campaign. Our finding that campaigns with endorsements are more successful than campaigns without endorsements is consistent with prior evidence from Kickstarter [34]. Finally, we confirm that active engagement with the crowd (e.g., in the form of lab notes) can increase fundraising success [33, 62] and that visual information such as videos is particularly beneficial [2, 23, 32].

Perhaps more importantly, we add to the crowdfunding literature by exploring aspects that have received less attention in prior work. Unlike prior studies, for example, we can observe creators’ substantive experience (proxied by position such as student versus professor) and show that experience has a *negative* relationship with funding success. This relationship is surprising, at least in light of the common belief that more experienced scientists are better able to identify important research questions and are better able to execute a given project [36, 71]. We noted some possible interpretations of this result in our earlier discussion. Either way, future research should examine how creators’ experience is related to funding success in other contexts, including settings where backers have a clear personal interest in substantive project outcomes (i.e., in rewards-based campaigns that pre-sell products).

Relatedly, we find that projects pursuing research versus development objectives are similarly likely to succeed. This result seems at odds with recent work showing that funding success on Kickstarter is higher for incremental than for radical innovations [15]. Although the research vs. development and radical vs. incremental distinctions are conceptually different, an intriguing conjecture for the different findings is that Kickstarter and Experiment.com have different types of “dominant crowds”: While typical backers on Kickstarter may be more concerned with the likelihood of success and the usefulness of project outcomes for their own needs, backers on Experiment.com may not expect to benefit personally from project results and may thus be willing to support also projects that are riskier or that promise general insights rather than tangible outcomes. More generally, our findings highlight the need for future research that examines the predictors of funding success across a range of different platforms and that explores the role of contingency factors such as the type of crowdfunding (e.g., rewards-based vs. donations) as well as the goals and motives of the typical backers.

Our results suggest a number of additional questions for future research. First, we observed that several characteristics of creators, projects, and campaigns are significant predictors of funding success, but we cannot establish the causal nature of these correlations. Experimental studies could provide further insights into underlying mechanisms and help identify tools that creators can use to improve the performance of their campaigns. Second, although the data allowed us to characterize creators and projects along a number of important dimensions, a better understanding is needed on which particular individuals select into using crowdfunding in the first place, and for which of their projects. Such work should explicitly consider which alternative funding mechanisms are available to scientists and may thus also inform us about the degree to which crowdfunding complements versus substitutes for existing mechanisms. Third, our data include no information about the backers, which is a challenge with crowdfunding research generally [20]. It would be interesting to know whether backers tend to come from particular parts of the general population (e.g., with respect to education, science background, or gender), and which creators are more successful in reaching beyond their friends and family. Fourth, future research is needed on why backers support scientific research projects, how their motivations differ from those of traditional funding agencies, and how backer motivations shape their interactions with creators. Fifth, future research is needed to measure the scientific output resulting from crowdfunded projects and to explore which projects are more likely to achieve their scientific objectives. A related question is whether crowdfunded projects allow researchers to subsequently obtain larger grants from traditional sources, helping bridge funding gaps for junior scientists or early-stage research. Sixth, crowdfunding opens a new channel of communication between scientists and the general public, similar to other emerging mechanisms such as citizen science. Future research could investigate the implications of such stronger interactions with the crowd in terms of the choice of research topics or the quality and impact of scientific work [69, 70]. Finally, crowdfunding of scientific research is a relatively new phenomenon and there will likely be changes with respect to many interesting aspects such as the creators seeking crowdfunding, platform types and infrastructure, awareness among the general public and the resulting supply of resources, and perhaps even the predictors of crowdfunding success. Future work tracing such developments over time using longitudinal data can provide important insights into crowdfunding specifically, but perhaps also more generally on the evolution of new institutions to support scientific research.

## Supporting information

S1 Fig Regions’ share of total funding and total number of funded projects, by funding source. (PDF)

S1 Table Selected correlations. (PDF)

S2 Table Regressions using team averages of creator characteristics. (PDF)

S3 Table Regressions excluding affiliation variables and using simple risk score. (PDF)
