# Supplementary material for: Crowdfunding scientific research: Descriptive insights and correlates of funding success
Source: PLoS One. 2019 Jan 4;14(1):e0208384. doi: 10.1371/journal.pone.0208384 (PMC6319731; doi:10.1371/journal.pone.0208384)
Supplement: S1 Fig — (PDF) [file pone.0208384.s001.pdf]

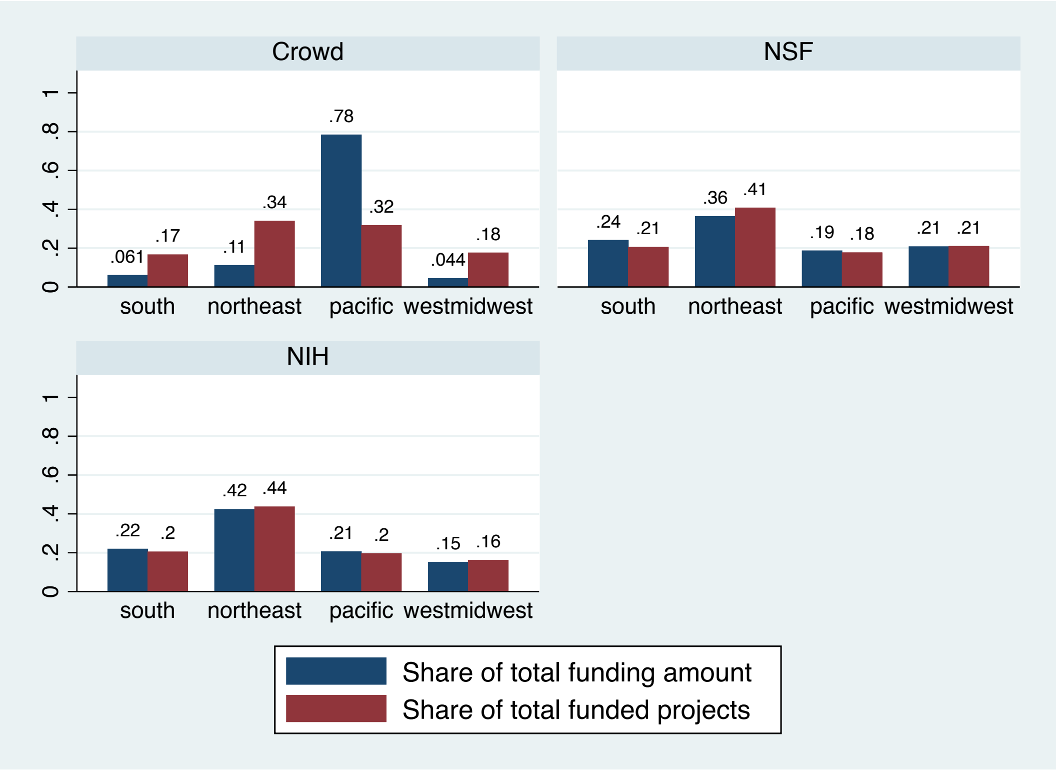

S1 Fig. Region's share of total funding and total number of funded projects, by funding source. NSF and NIH data pooled for years 2012-2015, from dellweb.bfa.nsf.gov and report.nih.gov/award/index.cfm.
