# Supplementary material for: Crowdfunding scientific research: Descriptive insights and correlates of funding success
Source: PLoS One. 2019 Jan 4;14(1):e0208384. doi: 10.1371/journal.pone.0208384 (PMC6319731; doi:10.1371/journal.pone.0208384)
Supplement: S1 Table — (PDF) [file pone.0208384.s002.pdf]

S1 Table. Selected correlations.

		1	2	3	4	5	6	7	8	9	10	11	12	13	14	15	16	17	18	19	20
**1**	Funded 01	1																			
**2**	Ln_amount raised	0.6530*	1																		
**3**	Press coverage 01	0.0863*	0.1131*	1																	
**4**	Below PhD/MD	0.1434*	0.0054	-0.0996*	1																
**5**	PhD/MD student	0.0462	0.0273	0.0014	-0.2840*	1															
**6**	Postdoc	0.0746*	0.0875*	0.0752*	-0.1182*	-0.1255*	1														
**7**	Assistant professor	-0.1007*	-0.0685	0.0639	-0.1770*	-0.1879*	-0.0782*	1													
**8**	Associate/full professor	-0.1201*	-0.0103	0.0960*	-0.2081*	-0.2210*	-0.0920*	-0.1377*	1												
**9**	Educational institution	0.0036	-0.0414	0.069	0.2575*	0.2734*	0.0979*	0.1704*	0.2003*	1											
**10**	Firm	-0.0803*	0.0146	-0.0435	-0.1233*	-0.1309*	-0.0545	-0.0816*	-0.0959*	-0.4789*	1										
**11**	Female	0.1371*	0.1183*	0.0658	0.0396	0.1229*	-0.0034	0.0374	-0.0842*	0.0647	-0.0426	1									
**12**	Ln_target	-0.2002*	0.2844*	0.1414*	-0.1812*	-0.1011*	0.031	0.0304	0.2171*	-0.0912*	0.1341*	-0.0669	1								
**13**	Goal: Research	0.017	0.0758*	0.1057*	-0.0777*	0.1626*	0.0623	0.0764*	0.0449	0.2288*	-0.062	0.1303*	0.0292	1							
**14**	Goal: Development	-0.0629	-0.0828*	-0.0584	0.0795*	-0.1022*	-0.0258	-0.0709	0.0108	-0.1034*	0.1189*	-0.0972*	0.0086	-0.6857*	1						
**15**	Risk score	-0.0850*	-0.0776*	0.0405	-0.0723	0.0194	-0.02	0.0743*	-0.0207	-0.0303	0.0571	0.0364	-0.0219	-0.009	0.0078	1					
**16**	Endorsement 01	0.1744*	0.2049*	0.0752*	-0.0114	0.0229	0.0095	0.031	-0.1035*	-0.04	-0.0156	0.0756*	0.0663	0.0712	-0.0852*	-0.0157	1				
**17**	Video 01	0.1850*	0.3122*	0.1310*	-0.022	0.0274	0.1059*	-0.0712	-0.0099	-0.0278	-0.006	-0.0228	0.1888*	0.0132	-0.0186	-0.0401	0.1601*	1			
**18**	Lab notes pre closing 01	0.3176*	0.4510*	0.1126*	-0.0318	0.0311	0.1040*	0.0157	-0.0278	0.0098	-0.07	0.0766*	0.0905*	0.0294	-0.0435	-0.0894*	0.1204*	0.2475*	1		
**19**	Rewards 01	0.1646*	0.1725*	0.0948*	-0.0137	0.0972*	0.0655	-0.0449	-0.0482	0.0033	-0.0425	0.0431	0.0075	0.0033	-0.0446	-0.0062	0.1537*	0.1414*	0.1144*	1	
**20**	No prior publications	0.0826*	-0.0494	-0.2110*	0.2717*	0.014	-0.0847*	-0.1302*	-0.1848*	-0.0297	-0.0848*	0.0337	-0.1825*	-0.1106*	0.0701	-0.047	-0.017	-0.0406	-0.0336	-0.0241	1
**21**	Creator count	0.0598	0.1266*	0.0528	-0.0379	-0.0508	0.0579	-0.0261	0.1566*	0.0442	-0.06	0.0293	0.1063*	-0.0962*	0.0784*	-0.0143	-0.0217	0.1022*	0.1106*	0.0044	0.0229

Note: *=sig. at 5%, first author observations only.
