# Supplementary material for: Crowdfunding scientific research: Descriptive insights and correlates of funding success
Source: PLoS One. 2019 Jan 4;14(1):e0208384. doi: 10.1371/journal.pone.0208384 (PMC6319731; doi:10.1371/journal.pone.0208384)
Supplement: S2 Table — (PDF) [file pone.0208384.s003.pdf]

S2 Table. Regressions using team averages of creator characteristics.

	Funded 01		(Ln) amount raised		(Ln) target		Press attention 01	
	1	2	3	4	5	6	7	8
	logit	logit	OLS	OLS	OLS	OLS	logit	logit
Share below PhD/MD	2.593**	2.818**	0.804*	0.760*	-1.033**	-0.930**	0.383*	0.506
	[0.916]	[1.113]	[0.313]	[0.299]	[0.127]	[0.133]	[0.154]	[0.219]
Share PhD/MD	1.307	1.038	0.680*	0.473+	-0.770**	-0.746**	0.644	0.745
	[0.456]	[0.397]	[0.301]	[0.280]	[0.130]	[0.130]	[0.252]	[0.313]
Share postdoc	2.117	1.262	0.797*	0.190	-0.491**	-0.634**	0.801	0.737
	[1.016]	[0.665]	[0.393]	[0.361]	[0.177]	[0.184]	[0.407]	[0.413]
Share assistant professor	0.791	0.571	0.214	-0.092	-0.500**	-0.532**	0.928	0.995
	[0.324]	[0.257]	[0.367]	[0.333]	[0.151]	[0.150]	[0.398]	[0.456]
Share employee	0.607	0.553	0.179	0.223	-0.748**	-0.673**	0.536	0.565
	[0.295]	[0.290]	[0.449]	[0.412]	[0.208]	[0.206]	[0.275]	[0.293]
Share individual/no affiliation	1.842	1.700	0.472	0.326	-0.771**	-0.707**	0.514	0.673
	[0.807]	[0.886]	[0.449]	[0.422]	[0.189]	[0.194]	[0.290]	[0.393]
Share firm	1.759	2.215	0.454	0.435	0.627**	0.548*	0.699	0.790
	[1.053]	[1.459]	[0.470]	[0.420]	[0.241]	[0.224]	[0.476]	[0.547]
Share other organization	2.184	2.239	0.471	0.291	0.332	0.308	0.726	0.891
	[1.043]	[1.189]	[0.415]	[0.371]	[0.233]	[0.228]	[0.416]	[0.485]
Share female	1.784**	1.757*	0.478**	0.374*	-0.009	-0.017	1.275	1.323
	[0.368]	[0.388]	[0.163]	[0.145]	[0.082]	[0.080]	[0.306]	[0.322]
Share gender N/A or unknown	0.622	0.554	-0.061	-0.090	0.334	0.329	0.571	0.716
	[0.324]	[0.312]	[0.443]	[0.432]	[0.251]	[0.254]	[0.496]	[0.552]
(Ln) target	0.665**	0.510**	0.673**	0.521**			1.561**	1.402**
	[0.067]	[0.061]	[0.083]	[0.081]			[0.184]	[0.168]
Objective: Research	omitted	omitted	omitted	omitted	omitted	omitted	omitted	omitted
Objective: Development	0.782	0.897	-0.340	-0.232	-0.070	-0.040	0.624	0.745
	[0.241]	[0.293]	[0.263]	[0.237]	[0.119]	[0.119]	[0.232]	[0.287]
Objective: Other	1.189	1.218	-0.109	-0.070	-0.169	-0.134	0.600	0.639
	[0.402]	[0.433]	[0.282]	[0.267]	[0.134]	[0.137]	[0.252]	[0.273]
Risk score	0.989	0.995	-0.008	-0.001	-0.003	-0.002	1.000	1.003
	[0.009]	[0.010]	[0.007]	[0.007]	[0.004]	[0.004]	[0.012]	[0.012]
Prior pubs: 1 listed		1.460		0.478*		0.087		3.752**
		[0.557]		[0.241]		[0.125]		[1.265]
Prior pubs: 2 listed		1.079		0.541*		0.008		3.906**
		[0.543]		[0.248]		[0.191]		[1.707]
Prior pubs: 3+ listed		0.777		-0.432		0.123		2.401*
		[0.300]		[0.311]		[0.139]		[1.058]
Prior pubs mentioned/linked		0.490*		-0.069		0.311*		1.113
		[0.166]		[0.223]		[0.128]		[0.411]
Endorsements 01		2.783**		0.603**		0.155		1.304
		[0.784]		[0.145]		[0.107]		[0.366]
Video 01		1.738**		0.484**		0.288**		1.778*
		[0.356]		[0.146]		[0.071]		[0.442]
Lab notes pre closing 01		3.930**		1.403**		0.130+		1.547+
		[0.869]		[0.164]		[0.075]		[0.384]
Reward 01		2.483*		0.578**		-0.020		1.167
		[0.908]		[0.176]		[0.107]		[0.360]
Press coverage 01		1.598+		0.022		0.216**		
		[0.391]		[0.164]		[0.082]		
Creator count	1.142	1.065	0.176**	0.114+	0.079*	0.064*	1.079	1.056
	[0.098]	[0.094]	[0.068]	[0.060]	[0.031]	[0.032]	[0.099]	[0.099]
Region fixed effects	incl.	incl.	incl.	incl.	incl.	incl.	incl.	incl.
Field fixed effects	incl.	incl.	incl.	incl.	incl.	incl.	incl.	incl.
Platform age	incl.	incl.	incl.	incl.	incl.	incl.	incl.	incl.
Constant	68.476**	276.137**	1.377	1.362	9.391**	9.042**	0.000**	0.000**
	[88.617]	[418.178]	[0.939]	[0.869]	[0.332]	[0.349]	[0.000]	[0.000]
Observations	721	721	721	721	721	721	721	721
df	31	40	31	40	30	39	31	39
Pseudo R-squared	0.186	0.284					0.113	0.165
R-squared			0.250	0.398	0.195	0.247		

Note: +=sig. at 10%, *=sig. at 5%, **=sig. at 1%. Robust standard errors in brackets. Odds ratios reported for logits (values <1 indicate a negative relationship).
