# Supplementary material for: Crowdfunding scientific research: Descriptive insights and correlates of funding success
Source: PLoS One. 2019 Jan 4;14(1):e0208384. doi: 10.1371/journal.pone.0208384 (PMC6319731; doi:10.1371/journal.pone.0208384)
Supplement: S3 Table — (PDF) [file pone.0208384.s004.pdf]

S3 Table. Regressions excluding affiliation variables and using simple risk score.

	Funded01	(Ln) raised	(Ln) target	Press 01	Funded01	(Ln) raised	(Ln) target	Press 01
	1	2	3	4	5	6	7	8
	logit	OLS	OLS	logit	logit	OLS	OLS	logit
Position: Below PhD/MD	4.054**	0.036	-0.885**	0.307**	2.851**	0.622*	-0.883**	0.478*
	[1.296]	[0.287]	[0.115]	[0.107]	[0.954]	[0.278]	[0.116]	[0.178]
Position: PhD/MD	2.389**	0.188	-0.649**	0.545+	1.807+	0.589*	-0.667**	0.690
	[0.732]	[0.267]	[0.115]	[0.179]	[0.579]	[0.255]	[0.115]	[0.239]
Position: Postdoc	3.789**	0.721*	-0.387*	1.024	3.302**	0.937**	-0.416*	1.231
	[1.578]	[0.296]	[0.161]	[0.437]	[1.394]	[0.293]	[0.161]	[0.526]
Position: Assistant professor	1.165	-0.196	-0.381**	0.894	0.965	0.048	-0.379**	1.029
	[0.422]	[0.334]	[0.131]	[0.318]	[0.355]	[0.339]	[0.132]	[0.381]
Position: Associate/Full professor	omitted	omitted	omitted	omitted	omitted	omitted	omitted	omitted
Position: Employee	1.713+	0.217	-0.382**	0.499*	0.875	-0.040	-0.787**	0.729
	[0.545]	[0.271]	[0.118]	[0.176]	[0.403]	[0.407]	[0.186]	[0.357]
Position: Individual/no affiliation	2.506*	-0.273	-0.746**	0.355+	1.904	0.268	-0.712**	0.530
	[1.082]	[0.394]	[0.185]	[0.193]	[0.821]	[0.429]	[0.184]	[0.300]
Position: Other position	2.973+	0.621	0.165	1.309	2.434	0.263	-0.001	1.532
	[1.942]	[0.774]	[0.408]	[1.158]	[1.561]	[0.666]	[0.363]	[1.522]
Affiliation: Educational institution					omitted	omitted	omitted	omitted
Affiliation: Firm					1.553	0.616	0.747**	0.619
					[0.877]	[0.441]	[0.229]	[0.398]
Affiliation: Other organization					2.273+	0.827*	0.489*	0.891
					[1.030]	[0.379]	[0.209]	[0.473]
Gender: Female	1.543*	0.445**	0.025	1.263	1.539*	0.386**	-0.005	1.281
	[0.281]	[0.151]	[0.074]	[0.267]	[0.288]	[0.144]	[0.076]	[0.276]
Gender: N/A or unknown	0.681	0.235	0.280	0.499	0.744	0.053	0.270	0.380
	[0.332]	[0.442]	[0.193]	[0.386]	[0.366]	[0.383]	[0.189]	[0.358]
(Ln) target					0.651**	0.639**		1.563**
					[0.065]	[0.081]		[0.186]
Objective: Research					omitted	omitted	omitted	omitted
Objective: Development					0.755	-0.369	-0.104	0.631
					[0.232]	[0.262]	[0.124]	[0.237]
Objective: Other					1.147	-0.152	-0.179	0.570
					[0.384]	[0.285]	[0.134]	[0.235]
Risk score simple					0.996	-0.008	-0.004	1.008
					[0.009]	[0.008]	[0.003]	[0.011]
Creator count	1.186*	0.218**	0.047	1.049	1.220*	0.196**	0.056+	1.048
	[0.097]	[0.074]	[0.032]	[0.092]	[0.105]	[0.070]	[0.032]	[0.096]
Region fixed effects	incl.	incl.	incl.	incl.	incl.	incl.	incl.	incl.
Field fixed effects	incl.	incl.	incl.	incl.	incl.	incl.	incl.	incl.
Platform age	incl.	incl.	incl.	incl.	incl.	incl.	incl.	incl.
Constant	1.105	7.633**	9.243**	0.011**	55.229**	1.664+	9.243**	0.000**
	[0.879]	[0.643]	[0.332]	[0.012]	[70.919]	[0.907]	[0.321]	[0.000]
Observations	721	721	721	721	721	721	721	721
df	26	26	26	26	32	32	31	32
Pseudo R-squared	0.162			0.0885	0.186			0.115
R-squared		0.164	0.173			0.255	0.192	

Note: +=sig. at 10%, *=sig. at 5%, **=sig. at 1%. Robust standard errors in brackets. Odds ratios reported for logits (values <1 indicate a negative relationship).
